# Associations Between Neonatal Brain Structure, the Home Environment, and Childhood Outcomes Following Very Preterm Birth

**DOI:** 10.1016/j.bpsgos.2021.05.002

**Published:** 2021-05-25

**Authors:** Lucy D. Vanes, Laila Hadaya, Dana Kanel, Shona Falconer, Gareth Ball, Dafnis Batalle, Serena J. Counsell, A. David Edwards, Chiara Nosarti

**Affiliations:** a Centre for the Developing Brain, School of Biomedical Engineering & Imaging Sciences, King’s College London, London, United Kingdom; b Department of Child and Adolescent Psychiatry, Institute of Psychiatry, Psychology and Neuroscience, King’s College London, London, United Kingdom; c Department of Forensic and Neurodevelopmental Science, Institute of Psychiatry, Psychology and Neuroscience, King’s College London, London, United Kingdom; d Developmental Imaging, Murdoch Children’s Research Institute, Melbourne, Victoria, Australia; e Department of Paediatrics, University of Melbourne, Melbourne, Victoria, Australia

**Keywords:** Parenting, Preterm birth, Preterm phenotype, Psychopathology, Stimulating home environment, Structural covariance networks

## Abstract

**Background:**

Very preterm birth is associated with an increased risk of childhood psychopathology and cognitive deficits. However, the extent to which these developmental problems associated with preterm birth are amenable to environmental factors or determined by neurobiology at birth remains unclear.

**Methods:**

We derived neonatal brain structural covariance networks using non-negative matrix factorization in 384 very preterm infants (median gestational age [range], 30.29 [23.57–32.86] weeks) who underwent magnetic resonance imaging at term-equivalent age (median postmenstrual age, 42.57 [37.86–44.86] weeks). Principal component analysis was performed on 32 behavioral and cognitive measures assessed at preschool age (*n* = 206; median age, 4.65 [4.19–7.17] years) to identify components of childhood psychopathology and cognition. The Cognitively Stimulating Parenting Scale assessed the level of cognitively stimulating experiences available to the child at home.

**Results:**

Cognitively stimulating parenting was associated with reduced expression of a component reflecting developmental psychopathology and executive dysfunction consistent with the preterm phenotype (inattention-hyperactivity, autism spectrum behaviors, and lower executive function scores). In contrast, a component reflecting better general cognitive abilities was associated with larger neonatal gray matter volume in regions centered on key nodes of the salience network, but not with cognitively stimulating parenting.

**Conclusions:**

Our results suggest that while neonatal brain structure likely influences cognitive abilities in very preterm children, the severity of behavioral symptoms that are typically observed in these children is sensitive to a cognitively stimulating home environment. Very preterm children may derive meaningful mental health benefits from access to cognitively stimulating experiences during childhood.

SEE COMMENTARY ON PAGE 87

Very preterm birth is associated with developmental problems including increased inattention, social and emotional difficulties (sometimes referred to as the preterm behavioral phenotype) (1), and deficits in executive function (2,3). However, there is substantial variation in the extent of impairment that preterm children experience, and there is insufficient knowledge of possible predictors and moderators of adverse outcomes in this population.

Premature exposure to the extrauterine environment is associated with macro- and microstructural brain maturational alterations (4, 5, 6, 7). Such alterations have been shown to be related to adverse childhood outcomes, particularly in the cognitive domain (10, 11, 12, 8, 9), and are also implicated in the heightened risk of developmental psychiatric disorders in preterm cohorts because many affected regions are involved in important aspects of mental functioning such as socioemotional processing (13,14), attention (15), and emotional regulation (16). Given the coordinated development of spatially disparate brain regions (17), coupled with an increasing appreciation of brain network dysfunction underlying psychopathology in general (18), it is important to elucidate neural underpinnings of adverse outcomes of preterm birth at the network level. A useful approach to explore structural brain networks is to assess anatomical covariance of regional volume across subjects, resulting in the delineation of structural covariance networks (SCNs). SCNs are thought to arise from coordinated maturation of distinct brain regions (17,19), show convergence with the brain’s intrinsic functional network architecture (20), and are altered in psychiatric disease (21, 22, 23). Altered structural covariance has been reported in preterm-born adolescents (24) and young adults (25). Notably, the association between low gestational age at birth (GA) and executive deficits was shown to be mediated by altered structural covariance in orbitofrontal, temporal, parietal, and subcortical regions in a large sample of adolescents (26). However, it is unclear to what extent SCNs emerging in the neonatal period might predict developmental outcomes after preterm birth.

The manner in which neurodevelopmental risk resulting from preterm birth unfolds individually is likely to be amenable to experiential factors, including parental behavior (27,28). It is also possible that those born preterm may benefit more strongly from an improved home environment than their term-born peers, as posited by theories of differential susceptibility (29,30). For example, parental responsivity and a highly stimulating home environment moderate the association between low birth weight on cognitive development (31) and attention-deficit/hyperactivity disorder (ADHD) symptoms (32). There is also evidence that effects of parenting on social and emotional outcomes may be mediated by their impact on cognitive development uniquely in preterm children (33). Promoting cognitive development in early childhood after preterm birth might be particularly important, given that cognitive delays could negatively affect social, emotional, and behavioral development (34, 35, 36). Cognitively stimulating parenting (indexing the extent of a child’s access to cognitively stimulating items and experiences at home) has been shown to promote school success in both term- and preterm-born children (37) and cognitive function in toddlers with congenital heart disease (38). However, it is not clear whether cognitively stimulating parenting also positively affects the mental health sequelae associated with preterm birth.

Environmental influences on neurodevelopmental outcomes after preterm birth are particularly important to understand because these insights can be exploited to inform the development of interventions and support measures. While it is clear that very-preterm-born infants are exposed to an increased risk of disrupted neurodevelopment, understanding how adverse effects of preterm birth can be mitigated by external factors is of paramount importance. Similarly, understanding the neural correlates of childhood outcomes that are less amenable to environmental factors is critical in facilitating early identification of individuals who may need greater support.

Here, we investigate relative effects of neonatal brain structure and cognitively stimulating parenting on behavioral outcomes in children born very preterm. By summarizing a range of outcomes spanning temperament, psychopathology, and cognitive functioning using principal component analysis (PCA), we aim to characterize the latent structure of behaviors, experiences, and symptoms typically observed in very preterm children. PCA allows for the identification of groups of symptoms and behaviors that tend to co-occur within subjects, resulting in orthogonal behavioral components that offer a more parsimonious description of all observed outcomes. We apply non-negative matrix factorization (NNMF) to voxelwise brain volumetric data collected at term-equivalent age to identify SCNs in the neonatal preterm brain and assess the effect of regional volumes of these networks, as well as cognitively stimulating parenting (37), on childhood outcomes.

## Methods and Materials

### Sample

Study participants were 511 very-preterm-born infants (birth at <33 weeks’ GA) enrolled in the Evaluation of Preterm Imaging study (ePrime, EudraCT: 2009-011602-42). Infants were recruited at birth in 2010–2013 from hospitals within the North and Southwest London Perinatal Network. Full details of the ePrime study can be found in Edwards *et al.* (39). Infants underwent magnetic resonance imaging (MRI) at term-equivalent age (38–44 weeks postmenstrual age [PMA]). Between the ages of 4 and 7 years, 251 children from the cohort underwent a neurodevelopmental follow-up assessment at the Centre for the Developing Brain, St Thomas’ Hospital, London. Written informed consent was obtained from participants’ caregiver(s) following procedures approved by the Stanmore Research Ethics Committee (14/LO/0677). The study was carried out in accordance with the Code of Ethics of the World Medical Association. We report findings based on 384 neonatal scans and 206 follow-up assessments. Combined data for both neonatal scans at childhood follow-up were available for 157 subjects.

### Perinatal and Demographic Data

Perinatal clinical and sociodemographic data were collected from the Standardized Electronic Neonatal Database. Parental postcode at the time of infant birth was used to derive an Index of Multiple Deprivation (IMD) score (Department for Communities and Local Government, 2011; https://tools.npeu.ox.ac.uk/imd/) as a measure of socioeconomic status. IMD is based on seven domains of deprivation within each neighborhood: income, employment, education, skills and training, health and disability, barriers to housing and services, and living environment and crime. Higher IMD values indicate higher deprivation.

### MRI Acquisition and Processing

Infants underwent MRI (T2-weighted turbo spin echo) at term-equivalent age. Scanner details and acquisition parameters can be found in the Supplement. Scans were classified by an experienced perinatal radiologist as containing major lesions (cystic periventricular leukomalacia, periventricular hemorrhagic infarction), minor (any other), or no lesions, and infants with major lesions were excluded from MRI analyses. For detailed processing procedures, see the Supplement. Deformation tensor fields (i.e., warps) from the nonlinear registration to a study-specific template were used to obtain a logarithm transformation of Jacobian determinant maps, reflecting local expansion/shrinkage of each voxel with respect to the template (40). Jacobian determinants did not include the affine registration component and are therefore corrected for global differences in head size. Log Jacobian maps were smoothed (4-mm full width at half maximum) and downsampled to 2-mm isotropic resolution. Only brain tissue voxels defined within the neonatal version of the Automated Anatomical Labeling atlas (41,42) were included in the analysis (resulting in 37,947 voxels). All voxel values were exponentiated before submitting to NNMF analysis to ensure non-negative input data.

### Outcome Assessments

Parents completed the following questionnaires indexing children’s temperament, behavior, and executive functioning: the Children’s Behavior Questionnaire Very Short Form (43); the Empathy Questionnaire (44); the Strengths and Difficulties Questionnaire (45); the ADHD-IV rating scale (46); the Social Responsiveness Scale (47); and the Behavior Rating Inventory of Executive Function, Preschool version (48). We used raw scores of the subscales for all parent-report measures. To assess general intelligence, children were administered the Wechsler Preschool and Primary Scale of Intelligence, Fourth Edition (WPPSI-IV) (49).

### Cognitively Stimulating Parenting

Parents completed a questionnaire adapted from the Cognitively Stimulating Parenting Scale reported in the study by Wolke *et al.* (37). It consists of 21 items included in the Home Observation for Measurement of the Environment Inventory (50) and was shown to have acceptable internal consistency (Cronbach α = 0.77) in children aged 6 years (37). Briefly, it assesses the availability and variety of experiences that promote cognitive stimulation in the home. This includes availability of educational toys, parental interactions such as teaching words or reading stories, and cognitively stimulating activities such as family excursions (see the Supplement and Table S1 for details on individual items).

### PCA of Outcome Data

Subscales of all outcome measures were included in PCA. Before conducting PCA, all scores were scaled to a mean of 0 and unit variance. All scores except for WPPSI scores (which were already normalized with respect to the relevant age group) were regressed against age at the follow-up assessment. Residuals from these regressions were used in subsequent PCA analyses. We used permutation testing and repeated split-half analyses to identify significant and reliable components. See the Supplement for details of this procedure.

### NNMF of Imaging Data

We used NNMF (see the Supplement for more information) to identify SCNs in the neonatal preterm brain in which regional brain volumes consistently covary across individuals (*N* = 384). NNMF is an unsupervised multivariate dimension reduction technique that is particularly suited for investigating brain structural covariance (26,51). In contrast to seed-based approaches (52), NNMF derives networks in a data-driven manner. Furthermore, because of non-negativity constraints of the decomposition, NNMF results in a parts-based representation of brain structure, which is readily interpretable. Compared with whole-brain voxelwise approaches, assessing brain structure at the SCN level not only considers biologically meaningful spatial patterns of covariation across the brain but also maximizes statistical power by reducing the number of comparisons.

NNMF factorized voxelwise Jacobian values (37,947 voxels × 384 subjects) into matrices *W* (37,947 voxels × *k* SCNs) and *H* (*k* SCNs × 384 subjects). The procedure to estimate the optimal rank *k* is detailed in the Supplement. Once the final NNMF (*k* = 15) had been estimated, we derived the weighted mean regional volumes for each of the *k* resulting SCNs for every subject. For each SCN, we calculated the mean log Jacobian for each subject with every voxel weighted by its relative contribution to the SCN (i.e., the voxelwise log Jacobian map multiplied by the column in *W* pertaining to that SCN, averaged for each subject). The resulting k SCN volumes per subject were used in subsequent analyses.

### Statistical Analysis

PCA identified three PCs, which were used as behavioral outcome variables throughout. First, we assessed the relationship between regional volumes of NNMF networks and behavioral outcome (*n* = 157). For each of the 15 SCNs, an omnibus test was conducted using multivariate linear regression, testing the effect of SCN volume on all three outcome PCs (PC1, PC2, and PC3) simultaneously, controlling for GA, PMA at scan, sex, and IMD. Where the multivariate effect of SCN volume on outcomes was significant at a corrected significance threshold of .05/15 = .003 (Bonferroni correction for 15 separate models; one for each SCN), individual follow-up linear regressions on each of the three PCs were performed to elucidate the nature and direction of associations, Bonferroni-corrected for multiple comparisons (i.e., three models).

Next, we assessed the relationship between behavioral outcome PCs and cognitively stimulating parenting (*n* = 206). An omnibus test was conducted, assessing the effects of cognitively stimulating parenting on all three outcome PCs simultaneously, controlling for GA, sex, and IMD. Significant effects were further investigated with individual follow-up linear regressions for each PC, Bonferroni-corrected for multiple comparisons.

Finally, for SCNs showing a significant effect on any outcome measure, we constructed a full model testing the combined additive effects of SCN volume and cognitively stimulating parenting on behavioral outcome PCs, controlling for GA, PMA at scan, sex, and IMD, to ascertain whether observed effects of SCN volume (or cognitively stimulating parenting) were significant over and above the effects of cognitively stimulating parenting (or SCN volume). We also tested whether the addition of an interaction between cognitively stimulating parenting and SCN volume significantly improved model fit for any behavioral outcome using likelihood ratio *F* tests. This tests explicitly whether parenting moderates an existing effect of SCN volume on behavior.

Analysis code is available at https://github.com/lucyvanes/preterm-outcomes.

### Sensitivity Analyses

We conducted several sensitivity analyses to ensure that observed effects were not driven by outliers or incidental sample characteristics. Analyses were repeated 1) after removing individuals from sets of twins and triplets at random; 2) after removing outliers (mean ± 3 × standard deviation) on any of the behavioral variables, SCN volumes, or cognitively stimulating parenting; 3) controlling for severity of brain lesions; and 4) controlling for maternal education (as an alternative measure of socioeconomic status to IMD) and maternal age.

## Results

Sample characteristics for the full sample and follow-up analysis subsamples can be found in Table 1. The complete follow-up sample did not differ from the baseline sample in terms of GA (*t*_539_ = 0.11, *p* > .05), PMA at scan (*t*_539_ = 0.25, *p* > .05), IMD (*t*_526_ = 1.72, *p* > .05), or sex distribution (χ^2^_1_ = 0.14, *p* > .05). Results of all sensitivity analyses can be found in the Supplement. In sum, direction and significance of effects reported in the following remained largely unchanged.

**Table 1:** Sociodemographic Sample Characteristics

Characteristics	Baseline MRI Sample, *n* = 384	Follow-up Behavioral Sample, *n* = 206	Complete MRI+Behavioral Sample, *n* = 157
GA at Birth, Weeks, Median [Range]	30.29 [23.57–32.86]	30.14 [23.86–32.86]	30.29 [24–32.86]
PMA at Scan, Weeks, Median [Range]	42.57 [37.86–44.86]	42.57 [38.29–52.86]	42.57 [38.29–44.86]
Female, *n* (%)	195 (50.8%)	102 (49.5%)	75 (47.7%)
IMD, Mean (SD)	20.02 (11.82)	18.27 (11.87)	18.08 (11.76)
Days in Intensive Care, Median [Range]	2 [0–52]	2 [0–54]	2 [0–51]
Minor Lesions, *n* (%)	218 (56.8%)	115 (55.8%)	94 (59.9%)
Major Lesions, *n* (%)	–	14 (6.8%)	–
Mother’s Age at Infant’s Birth, Years, Mean (SD)	32.84 (5.70)	33.83 (5.99)	33.92 (5.90)
Mother’s Age When Leaving FT Education, Years, *n* (%)			
≤16	39 (10.2%)	13 (6.3%)	8 (5.1%)
17–19	61 (15.9%)	29 (14.1%)	24 (15.3%)
≥19	272 (70.8%)	160 (77.7%)	122 (77.7%)
Still in FT	12 (3.1%)	4 (1.9%)	3 (1.9%)
Mother’s Ethnicity, *n* (%)			
White/White British	202 (52.6%)	120 (58.3%)	90 (57.3%)
Asian/Asian British	91 (23.7%)	44 (21.4%)	35 (22.3%)
Black/Black British	73 (19.0%)	32 (15.5%)	22 (14.0%)
Mixed race	7 (1.8%)	3 (1.5%)	3 (1.9%)
Other	6 (1.6%)	4 (1.9%)	4 (2.5%)
N/A	5 (1.3%)	3 (1.5%)	3 (1.9%)
Age at Follow-up Assessment, Years, Median [Range]	–	4.65 [4.19–7.17]	4.60 [4.19–7.17]
Cognitively Stimulating Parenting Scale, Mean (SD)	–	17.68 (2.43)	17.60 (2.39)

Age at follow-up assessment corrected for GA.

FT, full-time; GA, gestational age; IMD, Index of Multiple Deprivation; MRI, magnetic resonance imaging; N/A, data not available; PMA, postmenstrual age.

### PCs of Childhood Outcome

**Figure 1:**
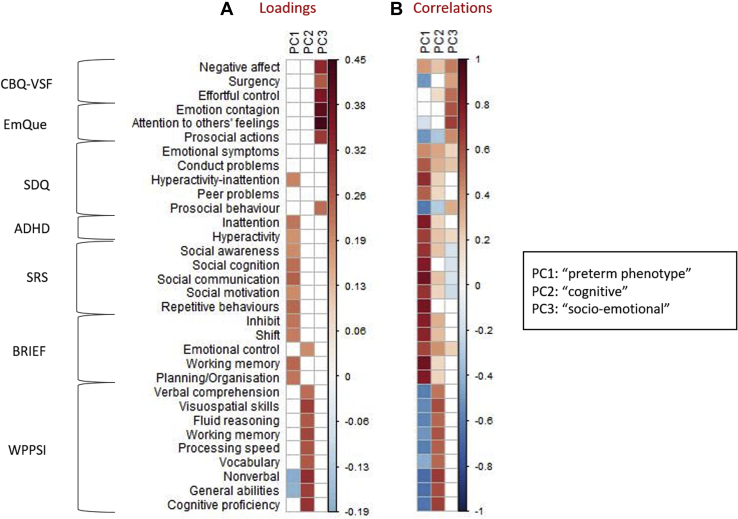
**(A)** Heatmap of loadings of each variable on principal component (PC) 1, PC2, and PC3, thresholded at 0.18 and **(B)** heatmap of significant correlations between each variable and PC1, PC2, and PC3. ADHD, ADHD-IV rating scale; BRIEF, Behavior Rating Inventory of Executive Function, Preschool version; CBQ-VSF, Children’s Behavior Questionnaire Very Short Form; EmQue, Empathy Questionnaire; SDQ, Strengths and Difficulties Questionnaire; SRS, Social Responsiveness Scale; WPPSI, Wechsler Preschool and Primary Scale of Intelligence.

PCA on outcome variables with permutation testing and repeated split-half analysis (see the Supplement for detailed results) identified three significant and reliable PCs (PC1–PC3), jointly explaining a cumulative 59% of total variance. Individual behavioral loadings as well as significant correlations with outcome variables for PC1–PC3 are depicted in Figure 1 and are listed in Table S2.

PC1 was driven by positive loadings of questionnaire subscales capturing ADHD symptoms (Strengths and Difficulties Questionnaire: hyperactivity-inattention; ADHD-IV: inattention; ADHD-IV: hyperactivity), autism spectrum symptoms (all subscales of the Social Responsiveness Scale), and executive deficits (Behavior Rating Inventory of Executive Function, Preschool version: inhibit, shift, working memory, and planning/organizing subscales), as well as negative loadings of scales capturing generalized cognitive abilities (nonverbal and general subscales of the WPPSI). Given the preponderance of ADHD and autism spectrum symptoms in conjunction with executive dysfunction typically observed in preterm cohorts, we termed this the “preterm phenotype” component. We interpret it as reflecting a measure of general (psychological and cognitive) dysfunction often observed in preterm children.

PC2 was driven by positive loadings of all subscales of the WPPSI (verbal comprehension, visuospatial skills, fluid reasoning, working memory, processing speed, vocabulary, non-verbal skills, general abilities, and cognitive proficiency). Notably, increased cognitive functioning reflected in this component does not appear to co-occur with decreased behavioral symptomatology (as is the case with PC1). Rather, loadings and correlations of symptom subscales tended to be positive rather than negative, although they did not on the whole load meaningfully onto this component. The exception to this is the emotional control subscale of the Behavior Rating Inventory of Executive Function, although its loading was less pronounced than that of WPPSI subscales (see Figure 1). This component therefore does not appear to reflect general behavioral (dys)function spanning cognition and psychopathology. Instead, we interpret this component to reflect purer aspects of cognition that are more independent from psychopathology and therefore termed it the “cognitive” component.

PC3 was driven by positive loadings of scales capturing childhood temperament (Children’s Behavior Questionnaire Very Short Form: negative affect, surgency, and effortful control) and empathy (Empathy Questionnaire: emotion contagion, attention to others’ feelings, and prosocial actions) as well as the Strengths and Difficulties Questionnaire prosocial behavior subscale. We termed this the “socioemotional” component. Owing to lower split-half reliability of PC3 compared with PC1 and PC2 (see the Supplement), we interpret this component with caution; however, we opted to retain it for further analysis to provide a more comprehensive summary of outcomes.

### NNMF-Derived SCNs

**Figure 2:**
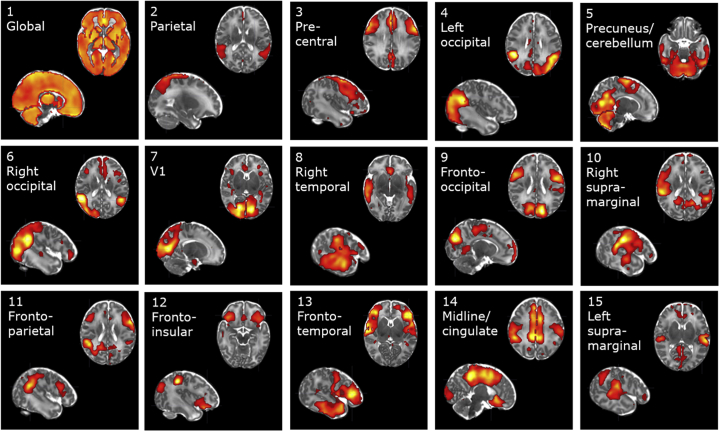
Structural covariance networks derived from non-negative matrix factorization on cortical and subcortical gray matter Jacobian volumes. For visualization purposes, voxelwise component weights were thresholded at 25% of the range for each network. Images are shown in radiological convention.

The rank selection procedure identified a 15-network solution as optimal (see the Supplement). All 15 SCNs are depicted in Figure 2. A brief summary of associations of SCN volumes with PMA and GA can be found in the Supplement.

### Association Between SCN Volumes and Outcomes

**Figure 3:**
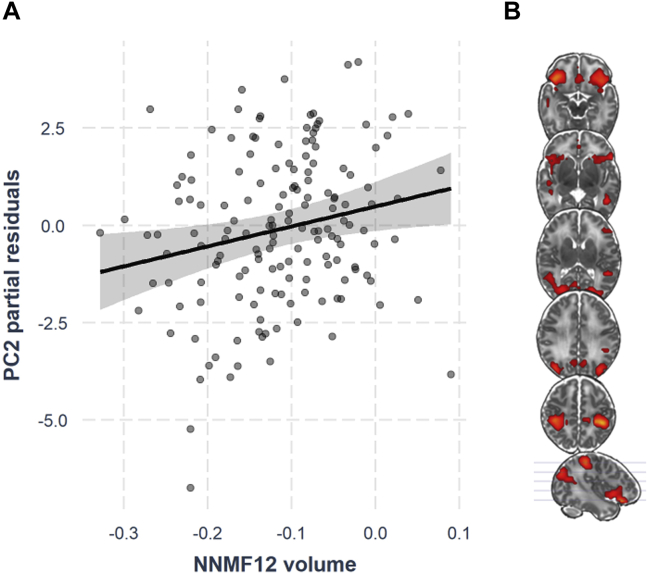
**(A)** Positive association between network 12 volume and cognitive component (principal component 2 [PC2]), adjusting for gestational age, postmenstrual age at scan, sex, and socioeconomic status. **(B)** Visualization of network 12. For visualization purposes, voxelwise component weights were thresholded at 25% of the range of values. Images are shown in radiological convention. NNMF, non-negative matrix factorization.

Omnibus multivariate regression analyses for each SCN, testing the effect of SCN volume on all three outcomes (PC1, PC2, and PC3) and controlling for GA, PMA, sex, and IMD indicated a significant effect of regional volume in SCN 12 (*p* = .003, passing Bonferroni correction), symmetrically encompassing anterior cingulate cortex, bilateral inferior frontal gyrus, bilateral insula, bilateral inferior parietal cortices, and bilateral middle occipital gyrus extending to precuneus. Follow-up univariate linear regressions revealed that this was driven by an (Bonferroni-corrected) association between greater volume of SCN 12 and greater expression of the cognitive component, PC2 (β = 5.12, *p* = .012) (Figure 3). A negative effect of SCN 12 volume on PC1 (preterm phenotype) did not survive Bonferroni correction (β = −8.15, *p* = .022), and the effect on PC3 (socioemotional) was nonsignificant (*p* > .05). Detailed results of the multivariate omnibus test and individual regressions, as well as sensitivity analyses, can be found in Table S3.

### Association Between Cognitively Stimulating Parenting and Childhood Outcomes

**Figure 4:**
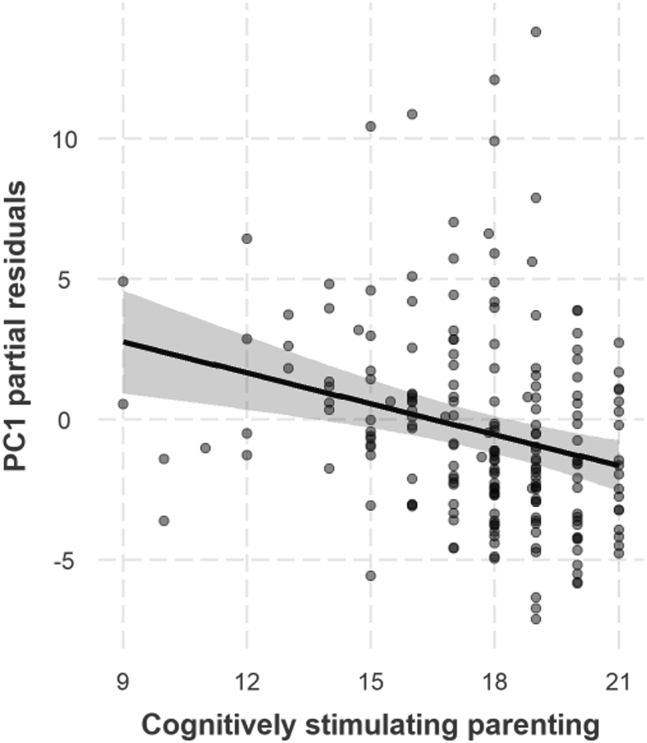
Negative association between cognitively stimulating parenting and preterm phenotype component (principal component [PC1]), adjusting for gestational age, sex, and socioeconomic status.

Multivariate regression of all three outcome variables (PC1, PC2, and PC3) on total Cognitively Stimulating Parenting scale score, controlling for GA, sex, and IMD, revealed a significant effect of cognitively stimulating parenting overall (*p* = .002). Follow-up univariate regression analyses revealed that this was driven by a (Bonferroni-corrected) significant negative effect of cognitively stimulating parenting on PC1 (β = −0.34, *p* < .001), indicating that a more stimulating home environment was associated with a reduction in symptom load on the preterm phenotype component (Figure 4). There was no effect of total cognitively stimulating parenting score on PC2 or PC3, all *p*s > .05. Detailed results and sensitivity analyses can be found in Table S4.

### SCN Volume, Cognitively Stimulating Parenting, and Outcomes

Finally, we constructed a full multivariate model of all three outcomes testing the effects of regional volume in SCN 12 and cognitively stimulating parenting score, controlling for GA, PMA, sex, and IMD. Both the effects of network volume (*p* = .004) and cognitively stimulating parenting (*p* = .047) were significant in the multivariate test. Follow-up linear regression analyses revealed that this was due to an effect of cognitively stimulating parenting on PC1 (preterm phenotype) (β = −0.26, *p* = .021) and an effect of SCN 12 volume on PC2 (cognitive) (β = 5.02, *p* = .016). The effects of cognitively stimulating parenting and SCN volume on PC3 (“socioemotional”) were nonsignificant. None of the models showed improvement by inclusion of an interaction term between SCN volume and cognitively stimulating parenting, as tested using likelihood ratio *F* tests, all *p*s > .05, indicating that cognitively stimulating parenting did not significantly moderate the effect of SCN 12 volume on behavioral outcomes.

## Discussion

We investigated the relative effects of neonatal brain structure and cognitively stimulating parenting on childhood outcomes in children born very preterm. We used PCA to characterize the latent structure of cognitive and behavioral outcomes at age 4–7 in this preterm cohort. The first identified component (PC1) reflected cognitive and behavioral features classically associated with very preterm birth (inattention, autism spectrum behaviors, and executive deficits), which varied as a function of the home environment: more cognitively stimulating parenting was associated with reduced symptom load on this component. A component reflecting improved cognitive performance (PC2) was, by contrast, predicted by greater regional volumes at term-equivalent age in an SCN encompassing bilateral inferior frontal gyrus, insula, and inferior parietal and middle occipital cortices. A third behavioral component (PC3) reflecting socioemotional problems was not related to either cognitively stimulating parenting or tissue volume in any of the 15 identified neonatal SCNs.

Our finding of an association between cognitively stimulating parenting and reduced preterm phenotype features is both intriguing and encouraging. It suggests that practical steps can be taken in the home environment to foster the development and well-being of very preterm children. Preterm-born individuals have been found to exhibit increased inattentive (nonhyperactive) ADHD symptoms (53) and, specifically, socializing problems associated with autism spectrum disorder (ASD) (54), both of which are associated with a detrimental impact on daily-life functioning (55,56). Inattention has been suggested to constitute the central behavioral deficit associated with preterm birth (57), which may even partially account for socializing difficulties observed in preterm children (57,58). Inattention and social difficulties are further associated with executive function deficits, which in turn mediate effects on daily-life functioning (55,59). PC1 appears to capture this behavioral tendency toward increased inattention, social dysfunction, and executive function deficits dimensionally in our cohort.

Parenting style is an important moderator of childhood cognitive and behavioral outcomes (60,61). Studies of cognitively stimulating parenting have focused on cognitive outcomes in childhood: intellectual and language abilities at age 5 (62) and school success from ages 6 to 13 years (37) were predicted by cognitively stimulating parenting in both preterm- and term-born individuals. Our findings expand on this by demonstrating that the benefits of cognitive stimulation for preterm children are not restricted to cognition, but rather affect behavioral aspects of development known to be most affected by preterm birth (encompassing both ADHD/ASD symptomatology and executive functioning) (1). In turn, improvement on this dimension is likely to translate into improved functioning in daily life, reflected in measures previously associated with cognitively stimulating parenting, such as school success (37). Notably, our sensitivity analyses showed that results held after controlling for severity of brain lesions, suggesting that the findings are applicable across the wider preterm population including those with no or minor injury, for whom prediction of outcome continues to be difficult.

It is important to note that while we did not find significant associations between neonatal regional brain volumes and PC1, this does not imply that this behavioral profile is altogether unrelated to neurobiology. Indeed, the causative pathway underlying both ADHD and ASD in preterm populations is generally thought to be more strongly characterized by neurobiological factors as a result of altered brain development following preterm birth (1). These neurodevelopmental alterations may be more readily detected in a direct comparison with term-born control subjects (63) and likely affect brain regions implicated in ADHD, anxiety, and ASD symptoms, including frontostriatal circuits and frontolimbic regions (14,64,65). However, our findings suggest that despite this likely neurodevelopmental disadvantage, preterm children can benefit from being provided with a cognitively stimulating home environment in the preschool years. There is evidence for reduced involvement in cognitive stimulation by parents of preterm children compared with parents of term-born children (62), suggesting that there is potential for targeted interventions here. Previous studies suggest that parental interventions for families of preterm and low-birth-weight infants can be effective in improving developmental outcomes such as behavioral problems (66) as well as attentional and autism spectrum symptoms (67), despite the strong neurobiological etiology of this type of symptomatology in this population.

In contrast to these findings, PC2, which we interpret as reflecting cognitive abilities more specifically (i.e., as being less related to behavioral outcomes), was predicted by larger neonatal regional tissue volumes in an SCN including frontoinsular, inferior parietal, and middle occipital cortices. The most pronounced involvement was that of bilateral anterior insula and inferior frontal gyrus, showing notable overlap with anterior portions of the salience network. The insula is one of the most densely connected regions of the developing brain (68); it is a major source of transient bursting events critical for brain maturation in preterm infants (69), and its connections are preferentially disrupted following preterm birth (70). The insula plays an important role in mediating among relevant networks to modulate behavior (71) and enable goal-directed cognitive processes (72,73). Coordination between regions belonging to the salience network, particularly anterior insula, and the default mode network has been shown to be disrupted in preterm-born adults (74), providing a potential mechanistic explanation for the emergence of cognitive deficits in this population.

Reduced tissue volumes have been observed in preterm individuals in predominantly temporal cortices, but extending to frontal, subcortical, and insular regions in neonates and children (75, 76, 77) as well as sensory and motor cortices in adolescents and adults (78,79). Furthermore, there is notable overlap between regions showing volumetric alterations and those associated with cognitive outcome across different age groups in preterm cohorts (13,79,80). For example, neonatal subcortical and insular volume predicted childhood working memory and mathematical skills in preterm, but not term-born, children (81). Interestingly, in young adults, lower IQ is associated with reduced white matter volumes beneath the left inferior frontal gyrus (79) and inferior frontal gyrus surface area (82), implicating that this as an important region underpinning general cognitive abilities in preterm individuals, in line with our findings in neonates.

A distinct advantage of the current procedure lies in the inclusion of several outcome measures spanning both cognitive and behavioral domains, from which we were able to derive components consisting of systematically covarying features within our cohort. This way, we were able to identify brain structural correlates of a specific cognitive component that is orthogonal to behavioral psychopathology observed in our sample. Our findings suggest that the effects of neuroanatomical disruption to an SCN encompassing inferior frontal, parietal, and insular cortices associated with cognitive outcome are already anchored in infancy. Note that our analysis did not show that the effect of SCN volume on behavior was moderated by cognitively stimulating parenting. It is possible that reduced neonatal regional volume of the insula in particular, as an important hub region for monitoring and switching (71), constrains the development of structural and functional within- and between-network connectivity necessary to flexibly adapt to changing cognitive demands. Finally, it is worth considering the possibility of common genetic effects underlying the observed association between volume in these regions and childhood cognition, given the role of genetic factors in precipitating preterm birth (83), modulating altered brain development in preterm infants (including that of insular connections) (70,84), and mediating developmental outcomes following prematurity (62,85).

Taken together, our results provide novel insights about the neonatal neurobiology underlying cognitive abilities in preterm children and the impact of the home environment on broader developmental psychopathology and executive deficits typically observed in this population. These findings carry important implications for the development of behavioral interventions in the care of preterm children. Future research can usefully address the mechanisms by which cognitively stimulating parenting fosters improvements in behavioral outcomes in preterm children, as well as potential interactions with neurobiological substrates of behavior not captured in this study.
