# Supplementary material for: Associations Between Neonatal Brain Structure, the Home Environment, and Childhood Outcomes Following Very Preterm Birth
Source: Biol Psychiatry Glob Open Sci. 2021 May 25;1(2):146–55. doi: 10.1016/j.bpsgos.2021.05.002 (PMC8367847; doi:10.1016/j.bpsgos.2021.05.002)
Supplement: Supplementary Materials [file mmc1.pdf]

# Associations Between Neonatal Brain Structure, the Home Environment and Childhood Outcomes Following Very Preterm Birth

## *Supplementary Information*

## Methods

### MRI acquisition and processing

Infants underwent MRI at term-equivalent age on a Philips 3T system (Philips Medical Systems, Best, The Netherlands) using an eight-channel phased array head coil. High resolution anatomical T2-weighted turbo spin echo was acquired with TR = 8670 ms, TE = 160 ms, flip angle 90°, slice thickness of 2 mm with 1 mm overlap, and in-plane resolution 0.86 × 0.86mm.

Images from successful T2 scans (N=486) were visually inspected to detect and exclude images with artefacts (N=10). Infants with major lesions (N=40) or PMA at scan ≥ 45 weeks (N=50) were excluded in order to minimise registration failures. After these exclusions, a further 2 subjects were removed due to processing failures. The processing pipeline included field bias correction and tissue type segmentation using the neonatal processing pipeline described in Makropoulos et al. (2014) (1). A study-specific template was generated from a subset of 161 participants using Advanced Normalization Tools (ANTS) software (2). Images were then registered to the study-specific template using multimodal Symmetric Normalisation (SyN) (2). Both T2-weighted images and T2-based tissue type segmentation were used as input modalities in order to improve image registration (3).

Deformation tensor fields (i.e. warps) from the non-linear registration were used to obtain a logarithm transformation of Jacobian determinant maps, reflecting local expansion/shrinkage of each voxel with respect to the template (4). Log Jacobian maps were spatially smoothed with a Gaussian kernel of 4mm full width at half maximum. In order to reduce computational load, maps were down-sampled to 2mm isotropic resolution. To further reduce the number of included voxels and aid in localisation of effects, only brain tissue voxels defined within the neonatal version of the Automated Anatomical Labelling (AAL) atlas (5, 6) were included in the analysis, resulting in an inclusion 37947 voxels. All voxel values were exponentiated before submitting to NNMF analysis in order to ensure non-negative input data.

## Cognitively Stimulating Parenting Scale

Parents completed a questionnaire adapted from the Cognitively Stimulating Parenting Scale reported in Wolke et al. (7). It consists of 21 items included in the Home Observation for Measurement of the Environment (HOME) Inventory (8) and has been shown to have acceptable internal consistency (Cronbach α = 0.77) (20). Briefly, the Cognitively Stimulating Parenting Scale assesses the availability and variety of experiences that promote cognitive stimulation in the home. 16 binary items capture the child's access to stimulating objects such as educational toys (6 items), parental interactions such as teaching numbers or colours (7 items), and parental behaviours such as reading habits (3 items). One item ("access to cassette player") was adapted to reflect technological advances appropriate at time of testing ("access to cassette/CD/DVD player"). Five additional Likert-scale items assess the frequency of cognitively stimulating experiences such as family excursions (4 items) and number of books in the home (1 item). All questionnaire items can be found in Supplemental Table 1. Yes/No items were scored as 1/0, and Likert-scale items were scored as 0 for answers 0-3 and as 1 for answers 4-6. A total sum score was calculated from all items (minimum 0, maximum 21).

## Principal component analysis (PCA)

Significance of the principal components (PCs) was assessed using permutation testing. For each of 5000 permutations, the subject order for each column was randomly shuffled and PCA recomputed, saving the proportion of variance explained by each permutation PC, thereby establishing the null model. Original PCs were deemed significant if the proportion of variance they explained exceeded chance ($p < .05$ across 5000 permutations).

In order to assess which of the significant PCs was consistent across independent sub-samples, we conducted a split-half replication. For each of 1000 iterations, we randomly split the sample into two non-overlapping sets of subjects and re-computed the PCA in each sub-sample. Correlations between the PC loadings from the two PCA solutions were evaluated for reproducibility, and significance of PCs for each sub-sample was again assessed using permutation testing as described above.

To assess meaningful contribution of individual variables to the identified PCs, we defined the loading threshold as $\sqrt{\frac{1}{N_{variables}}} = 0.18$, equivalent to the loading value if all 32 variables included in the PCA loaded equally onto the same component (since the sum of squares of all loadings for one PC must sum to 1). Loadings greater than this value were considered to be

meaningful. We also inspected correlations between PC loadings and original input variables (outcome assessments) to aid interpretation of the components.

## Non-negative Matrix Factorisation

Non-negative matrix factorisation (NNMF) is an unsupervised multivariate analysis technique which aims to obtain a low-rank approximation of high-dimensional data. NNMF decomposes a non-negative input matrix into the product of two low-rank factor matrices ($W$ and $H$). Due to non-negativity constraints imposed on each element of the resulting matrices, this procedure results in a parts-based representation of the data, whereby the parts are additively combined to reproduce the original input. This is a particularly advantageous property of NNMF as it increases interpretability and specificity of the resulting components compared to other dimension reduction techniques. It is especially suited to identifying components of covariance within data that is inherently non-negative, such as brain structural measures (9).

Here, we aimed to identify structural networks in the neonatal preterm brain in which regional brain volumes co-vary across individuals in a consistent way (N=384). We thus used a matrix containing voxelwise jacobian values (dimensions: 37947 voxels × 384 subjects) as input to the NNMF algorithm, resulting in a factorisation into a matrix $W$ (dimensions: 37947 voxels × $k$ networks), representing the individual contribution of each voxel to each of $k$ networks, and a matrix $H$ (dimensions: $k$ networks × 384 subjects), containing subject-specific scores for each of $k$ networks. The procedure to estimate the optimal rank $k$ is detailed in the following.

## Rank estimation procedure for Non-negative Matrix Factorisation (NNMF)

First, we ran NNMF on the original data for ranks 2 to 20, in steps of 1. NNMF was run 50 times for each rank, each time randomly masking 20% of values within the input matrix. Next, we randomly permuted the elements within each column of the input matrix, and repeated the NNMF estimation of this permuted dataset for ranks 2 to 20 a total of 50 times each, again masking a random subset of 20% of elements each time. For each NNMF estimation (both on the original and permuted datasets), we calculated the reconstruction error for each solution, defined as the Frobenius norm between the input data matrix (original or permuted) and the resulting NNMF approximation ($W \times H$). Additionally, we calculated the root mean square error (RMSE) for the 20% held-out datapoints as well as the included datapoints for each estimation.

The reconstruction error is expected to decrease as a function of increasing rank $k$, both for the original data and randomly permuted data. We therefore aimed to ascertain the optimal

rank *k* at which the decrease in reconstruction error for the original data no longer exceeds the decrease in reconstruction error for the permuted data (i.e., the point at which the decrease in error is no more substantial than the decrease observed in random data). For this, we performed a t-test at each rank comparing the *gradient* of the reconstruction error (capturing the decrease in error) for the original data and the permuted data (each consisting of 50 runs at that rank), and chose the highest rank at which this difference between original and permuted data was significant.

NNMF was run in R using the NNLM and NMF packages. NNLM was used (with initialisation based on random seeding) during the rank optimisation procedure, as it allows for individual matrix elements to be masked during approximation. NMF was used for the final estimation of structural covariance networks, using the previously ascertained optimal rank (with initialisation based on non-negative double singular value decomposition [NNDSVD] and no data held out).

## Results

### Principal components of childhood outcomes

PCA on outcome variables with permutation testing identified four significant PCs (PC1-PC4) in the full sample, explaining a cumulative 64% of total variance. Further permutation testing showed PC1 and PC2 to be consistently significant in both halves across 1000 split-half analyses (100% of split-halves). PC3 was significant in both halves in 62.8% of split-half analyses, and significant in only one half in a further 37.1%. PC4 was inconsistently significant (both halves: 0.1%; one half: 45.3% of analyses). The mean correlation between loadings in the two halves were high for PC1 (R = .98) and PC2 (R = .85), medium for PC3 (R = .46) and low for PC4 (R = -.03). We therefore retained PC1, PC2, and PC3 for further analysis, jointly explaining a cumulative proportion of 59% of total variance. Individual behavioural loadings (thresholded at 0.18) as well as significant correlations with outcome variables for PC1-PC3 are depicted in Figure 1. Numerical values of all loadings can be found in Supplementary Table S1.

### Results of rank selection for NNMF

As expected, reconstruction error decreased as a function of rank both for the original and permuted data (Figure S1A).

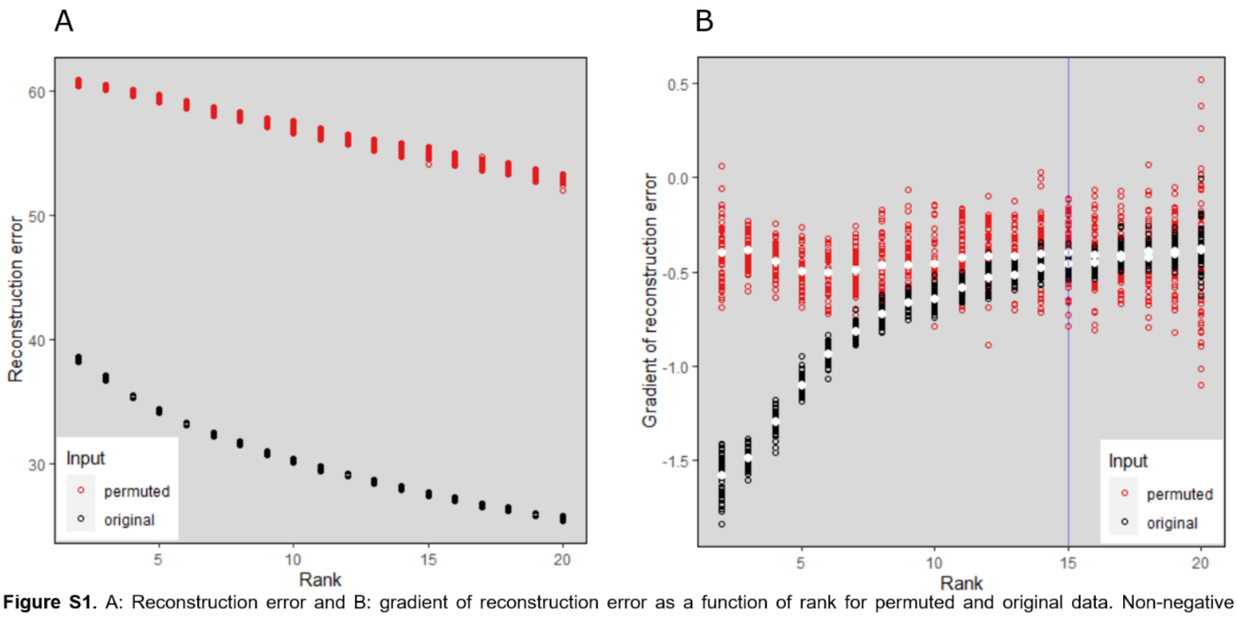


**Figure S1.** A: Reconstruction error and B: gradient of reconstruction error as a function of rank for permuted and original data. Non-negative matrix factorisation was estimated 50 times at each rank while randomly holding out 20% of matrix elements. Blue vertical line depicts highest rank (k=15) at which there was a significant difference between the means (depicted in white) of the reconstruction error gradients between permuted and original data.

Figure S1B shows the superimposed gradients of the reconstruction errors for original and permuted data, each estimated 50 times under 20% Wold holdouts. The difference between mean reconstruction error gradients was significant for ranks 1-15 and non-significant thereafter, indicating that at ranks 16 and above the information gained by adding one component no longer exceeds that gained in random data. Inspection of the gradient of RMSE for included (training) and held-out (test) datapoints also showed a levelling-out at around 15 networks (Figure S2). We therefore used the 15-network solution for all further analyses.

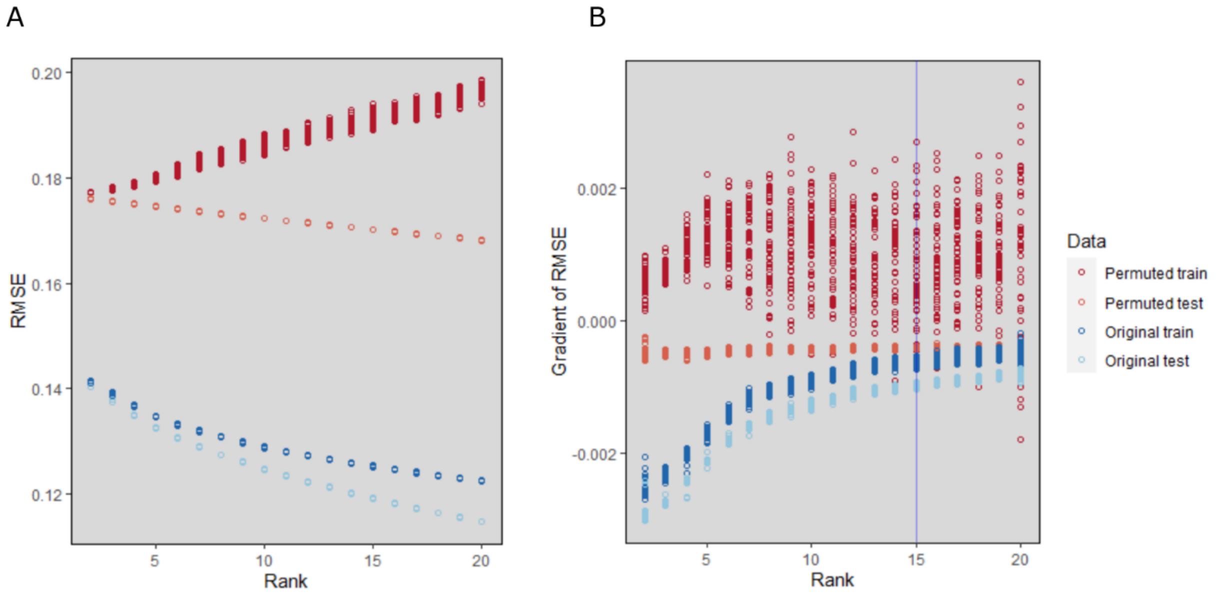


**Figure S2.** A: Root mean square error (RMSE) and B: gradient of RMSE as a function of rank for permuted and original train and test data. Non-negative matrix factorisation was estimated 50 times at each rank while randomly holding out 20% of matrix elements (test data). Blue vertical line depicts highest rank (k=15) at which there was a significant difference between the means of the reconstruction error gradients between permuted and original data.

## Effect of PMA and GA on structural covariance networks

There was a significant positive effect of PMA (controlling for GA) on regional volume in 7 networks (2, 3, 5, 8, 11, 13, and 14, all *p*s < .05), and a significant positive effect of GA (controlling for PMA) on regional volume in 10 networks (1, 2, 5, 6, 8, 10, 11, 12, 13, and 15, all *p*s < .05). Intriguingly, there was a significant negative effect of GA on volume in network 3 ($p < .001$), a predominantly sensorimotor network.

## Sensitivity analyses

### Removal of twins and triplets

We repeated all analyses after removing possible effects of the high proportion of twin and triplet sets in our data. Where a complete twin set, or at least two triplets from the same triplet set were present, we removed one twin or triplet (or two triplets, in the case of complete triplet sets) at random, and repeated all analyses. Before testing the effect of SCN volumes on behavioural outcome PCs, we removed 13 individual twins and 3 individual triplets, resulting in a total sample of N=140 (down from N=157) individuals. Controlling for multiple comparisons (15 models), SCN 12 was, again, the only SCN showing a significant multivariate effect on behavioural outcomes ($p < .001$), which was driven by its univariate effect on PC2 (“cognitive” component) ($\beta = 5.58$, $p = .007$) (see Table S3).

Before testing the effect of cognitively stimulating parenting on behavioural outcomes, we removed 23 individual twins and 4 individual triplets, repeating the analysis on N=175 (down from N=206) individuals. Multivariate and univariate effects are found in Table S4, showing that a multivariate effect of cognitively stimulating parenting was driven by its effect on PC1 ("preterm phenotype"), in line with the main analysis.

**Removal of outliers**

We repeated the analysis after removing subjects who had values ± 3 standard deviations from the mean for cognitively stimulating parenting (N=4), PC1 (N=5), PC2 (N=0), PC3 (N=1), or any of the SCN volumes (N=10).

After multiple comparison correction (for 15 models, one for each SCN), SCN 12 was, again, the only SCN showing a multivariate effect on behavioural outcomes ($p$ = .004; marginally significant at a corrected $p$-threshold of 0.05/15=.003), which was driven by its univariate effect on PC2 ("cognitive" component) ($\beta$ = 5.10, $p$ = .020) (see Table S3). Multivariate and univariate effects of cognitively stimulating parenting on behavioural outcomes are found in Table S4, showing that a multivariate effect of cognitively stimulating parenting ($p$ < .001) was driven by its effect on PC1 ("preterm phenotype"), ($\beta$ = -0.39, $p$ < .001), in line with the main analysis. The effect of cognitively stimulating parenting on PC3 ("socio-emotional") also became significant, ($\beta$ = --0.11, $p$ = .015).

**Controlling for lesions**

We repeated the analysis controlling for severity of brain lesions (major / minor / absent). As infants with major brain lesions were removed from analyses assessing brain-behaviour relationships, lesion severity was coded as minor vs absent in these analyses.

After multiple comparison correction (for 15 models, one for each SCN), SCN 12 was, again, the only SCN showing a significant multivariate effect on behavioural outcomes ($p$ < .001), which was driven by its univariate effect on PC2 ("cognitive" component) ($\beta$ = 5.18, $p$ = .011) (see Table S3). Multivariate and univariate effects of cognitively stimulating parenting on behavioural outcomes are found in Table S4, showing that a multivariate effect of cognitively stimulating parenting ($p$ = .002) was driven by its effect on PC1 ("preterm phenotype"), ($\beta$ = -0.34, $p$ < .001), in line with the main analysis.

In addition, we re-ran the analysis testing an effect of cognitively stimulating parenting on behavioural outcomes after excluding those participants with major lesions (i.e., the same subjects removed from all MRI analyses). In line with the main analysis, there was a significant multivariate effect of cognitively stimulating parenting ($p$ = .008), which was driven by its effect

on PC1 (”preterm phenotype”), ($\beta$ = -0.31 $p$ = .003), suggesting that the effect is not biased by those individuals who suffered major lesions at birth.

**Controlling for maternal age and education**

Maternal education, rather than IMD, could be studied as a proxy for socio-economic status. We therefore repeated our analysis controlling for maternal education instead of IMD. We also controlled for maternal age in this model. Maternal education was captured in terms of the age at which the mother left full-time education (16 or younger / 17 to 19 / 19 or older / Still in full-time education).

Of note, cognitively stimulating parenting was positively correlated with maternal age (R = 0.24, p < .001), and marginally significantly associated with maternal education (non-parametric Kruskal-Wallis test due to unequal sample size, $\chi(3) = 7.67$, p = .053), with more cognitively stimulating parenting reported by mothers who left full-time education at age 19 or older compared to those who left education aged 17-19 (W = 1685.5, p = 0.018), and marginally more compared to those who left education aged 16 or younger (W = 686.5, p = .097).

After multiple comparison correction (for 15 models, one for each SCN), SCN 12 was, again, the only SCN showing a significant multivariate effect on behavioural outcomes ($p$ = .003), which was driven by its univariate effect on PC2 (“cognitive” component) ($\beta$ = 6.04, $p$ = .005) (see Table S3). Multivariate and univariate effects of cognitively stimulating parenting on behavioural outcomes are found in Table S4, showing that a multivariate effect of cognitively stimulating parenting ($p$ = .013) was driven by its effect on PC1 (“preterm phenotype”), ($\beta$ = -0.26, $p$ < .010), in line with the main analysis.

*Table S1.* Items of the Cognitively Stimulating Parenting scale

| **Item** | **Response** | **Scoring** |
|---|---|---|
| 1. Availability of toys that teach colours and shapes | Yes/No | 1/0 |
| 2. Availability of cassette/DVD player | Yes/No | 1/0 |
| 3. Availability of fine-motor toys such as LEGO, colouring books, or arts and crafts materials | Yes/No | 1/0 |
| 4. Availability of toys that stimulate number knowledge | Yes/No | 1/0 |
| 5. Does the child own a musical instrument? | Yes/No | 1/0 |
| 6. Availability of ≥10 children's books | Yes/No | 1/0 |
| 7. Teaching of animal names | Yes/No | 1/0 |
| 8. Teaching of alphabet | Yes/No | 1/0 |
| 9. Teaching of colours | Yes/No | 1/0 |
| 10. Teaching of shapes | Yes/No | 1/0 |
| 11. Teaching of numbers | Yes/No | 1/0 |
| 12. Teaching of spatial relations and concepts | Yes/No | 1/0 |
| 13. Teaching of words | Yes/No | 1/0 |
| 14. Do parents read in free time? | Yes/No | 1/0 |
| 15. Do parents read daily newspaper? | Yes/No | 1/0 |
| 16. Do parents regularly read magazines? | Yes/No | 1/0 |
| 17. Reading to child or telling stories | 7-point Likert | 0-3=0, 4-6=1 |
| 18. Number of books at home | 7-point Likert | 0-3=0, 4-6=1 |
| 19. Frequency of family trips | 7-point Likert | 0-3=0, 4-6=1 |
| 20. Frequency of big trips or family vacations | 7-point Likert | 0-3=0, 4-6=1 |
| 21. Frequency of museum visits | 7-point Likert | 0-3=0, 4-6=1 |

*Table S2.* Component loadings of all behavioural outcome variables on P1, PC2, and PC3

| | PC1 | PC2 | PC3 |
|---|---|---|---|
| **CBQ-VSF** | | | |
| Negative affect | 0.112763 | 0.12327 | **0.330561** |
| Surgency | -0.14566 | -0.04868 | **0.253197** |
| Effortful control | 0.022616 | 0.067635 | **0.360155** |
| **Empathy Questionnaire** | | | |
| Emotion contagion | 0.038099 | -0.03576 | **0.411038** |
| Attention to others' feelings | -0.0475 | -0.01101 | **0.448563** |
| Prosocial actions | -0.14427 | -0.14541 | **0.303816** |
| **SDQ** | | | |
| Emotional symptoms | 0.124586 | 0.172112 | 0.138685 |
| Conduct problems | 0.1599 | 0.145571 | 0.176948 |
| Hyperactivity-inattention | **0.204221** | 0.103478 | 0.091929 |
| Peer problems | 0.157003 | 0.082958 | 0.06606 |
| Prosocial behaviour | -0.17308 | -0.13983 | **0.22814** |
| **ADHD** | | | |
| Inattention | **0.222404** | 0.095837 | 0.029214 |
| Hyperactivity | **0.183019** | 0.124954 | 0.121861 |
| **SRS** | | | |
| Social awareness | **0.196829** | 0.121195 | -0.101 |
| Social cognition | **0.226071** | 0.034558 | -0.10467 |
| Social communication | **0.242273** | 0.119338 | -0.10548 |
| Social motivation | **0.193491** | 0.092514 | -0.15326 |
| Repetitive behaviours | **0.235623** | 0.049635 | -0.03078 |
| **BRIEF-P** | | | |
| Inhibit | **0.220572** | 0.147286 | 0.092888 |
| Shift | **0.211082** | 0.134647 | 0.016528 |
| Emotional control | 0.178686 | **0.193801** | 0.151805 |
| Working memory | **0.239692** | 0.079265 | -0.01582 |
| Planning/Organisation | **0.223437** | 0.093154 | 0.031522 |
| **WPPSI** | | | |
| Verbal comprehension | -0.16771 | **0.232005** | -0.00856 |
| Visuospatial skills | -0.15439 | **0.289252** | 0.010025 |
| Fluid reasoning | -0.16464 | **0.255996** | -0.07012 |
| Working memory | -0.14391 | **0.284201** | -0.0172 |
| Processing speed | -0.16903 | **0.267851** | -0.04591 |
| Vocabulary | -0.12182 | **0.25968** | 0.060312 |
| Nonverbal | **-0.19329** | **0.320008** | -0.02188 |
| General abilities | **-0.18577** | **0.295253** | -0.0161 |
| Cognitive proficiency | -0.17775 | **0.310677** | -0.03945 |

*Table S3.* Results of multivariate and univariate regression analyses of childhood outcomes on neonatal structural covariance network (SCN) volumes

| | | *Univariate* | | | | | | | | |
|---|---|---|---|---|---|---|---|---|---|---|
| | | ***PC1*** | | | ***PC2*** | | | ***PC3*** | | |
| *Predictor* | *Multivariate F(df)* | *β* | *SE* | *p* | *β* | *SE* | *p* | *β* | *SE* | *p* |
| **Main analysis** | | | | | | | | | | |
| **SNC 1** | | | | | | | | | | |
| GA | 0.88(3,149) | -0.03 | 0.13 | .807 | 0.04 | 0.07 | .578 | 0.00 | 0.05 | .987 |
| Sex | 4.16(3,149) | 1.05 | 0.55 | .061 | 0.40 | 0.32 | .215 | -0.56 | 0.22 | .012 |
| PMA | 1.78(3,149) | 0.02 | 0.20 | .933 | -0.17 | 0.11 | .133 | -0.16 | 0.08 | .041 |
| IMD | 3.65(3,149) | 0.04 | 0.02 | .096 | -0.04 | 0.01 | .010 | 0.00 | 0.01 | .926 |
| Volume | 3.41(3,149) | -16.42 | 7.47 | .030 | 7.76 | 4.29 | .073 | 3.55 | 2.97 | .234 |
| **SNC 2** | | | | | | | | | | |
| GA | 0.83(3,149) | -0.10 | 0.12 | .400 | 0.07 | 0.07 | .326 | 0.01 | 0.05 | .806 |
| Sex | 4.13(3,149) | 1.03 | 0.56 | .067 | 0.40 | 0.32 | .209 | -0.56 | 0.22 | .013 |
| PMA | 1.76(3,149) | -0.02 | 0.20 | .910 | -0.18 | 0.12 | .124 | -0.17 | 0.08 | .036 |
| IMD | 3.47(3,149) | 0.04 | 0.02 | .104 | -0.04 | 0.01 | .005 | 0.00 | 0.01 | .761 |
| Volume | 0.72(3,149) | 2.20 | 2.81 | .435 | 1.53 | 1.61 | .342 | 0.97 | 1.11 | .380 |
| **SNC 3** | | | | | | | | | | |
| GA | 0.84(3,149) | -0.21 | 0.14 | .126 | 0.12 | 0.08 | .128 | 0.00 | 5.44 | .999 |
| Sex | 4.13(3,149) | 0.86 | 0.56 | .128 | 0.47 | 0.32 | .145 | -0.58 | 2.24 | .011 |
| PMA | 1.74(3,149) | 0.06 | 0.20 | .761 | -0.19 | 0.12 | .102 | -0.15 | 8.02 | .061 |
| IMD | 3.56(3,149) | 0.04 | 0.02 | .071 | -0.04 | 0.01 | .007 | 0.00 | 9.49 | .873 |
| Volume | 1.77(3,149) | -6.66 | 3.67 | .072 | 2.86 | 2.11 | .178 | -0.85 | 1.46 | .561 |
| **SNC 4** | | | | | | | | | | |
| GA | 0.85(3,149) | -0.09 | 0.12 | .480 | 0.07 | 0.07 | .328 | 0.01 | 0.05 | .840 |
| Sex | 4.17(3,149) | 1.19 | 0.57 | .040 | 0.36 | 0.33 | .271 | -0.61 | 0.22 | .007 |
| PMA | 1.76(3,149) | 0.00 | 0.20 | .999 | -0.16 | 0.11 | .156 | -0.16 | 0.08 | .045 |
| IMD | 3.51(3,149) | 0.05 | 0.02 | .057 | -0.04 | 0.01 | .007 | 0.00 | 0.01 | .776 |
| Volume | 1.25(3,149) | -3.74 | 2.83 | .187 | 0.99 | 1.62 | .542 | 1.29 | 1.11 | .249 |
| **SNC 5** | | | | | | | | | | |
| GA | 0.84(3,149) | -0.13 | 0.13 | .304 | 0.09 | 0.07 | .252 | 0.03 | 0.05 | .558 |
| Sex | 4.15(3,149) | 1.05 | 0.56 | .063 | 0.39 | 0.32 | .221 | -0.57 | 0.22 | .011 |
| PMA | 1.75(3,149) | -0.03 | 0.20 | .898 | -0.15 | 0.12 | .185 | -0.15 | 0.08 | .066 |
| IMD | 3.49(3,149) | 0.04 | 0.02 | .114 | -0.04 | 0.01 | .012 | 0.00 | 0.01 | .976 |
| Volume | 0.74(3,149) | 3.01 | 3.50 | .392 | -1.11 | 2.00 | .582 | -1.39 | 1.38 | .314 |
| **SNC 6** | | | | | | | | | | |
| GA | 0.88(3,149) | -0.08 | 0.13 | .514 | 0.03 | 0.07 | .622 | 0.00 | 0.05 | .939 |
| Sex | 4.13(3,149) | 1.06 | 0.56 | .062 | 0.34 | 0.31 | .280 | -0.57 | 0.22 | .010 |
| PMA | 1.80(3,149) | 0.02 | 0.20 | .926 | -0.21 | 0.11 | .069 | -0.17 | 0.08 | .034 |
| IMD | 3.64(3,149) | 0.04 | 0.02 | .069 | -0.04 | 0.01 | .003 | 0.00 | 0.01 | .808 |
| Volume | 3.03(3,149) | -2.41 | 3.51 | .493 | 5.35 | 1.96 | .007 | 1.45 | 1.38 | .296 |

| | | | | | | | | | | |
|---|---|---|---|---|---|---|---|---|---|---|
| **SNC 7** | | | | | | | | | | |
| GA | 0.86(3,149) | -0.10 | 0.13 | .410 | 0.09 | 0.07 | .212 | 0.03 | 0.05 | .494 |
| Sex | 4.16(3,149) | 1.04 | 0.56 | .068 | 0.39 | 0.32 | .225 | -0.57 | 0.22 | .009 |
| PMA | 1.83(3,149) | 0.00 | 0.20 | .989 | -0.16 | 0.11 | .174 | -0.15 | 0.08 | .055 |
| IMD | 3.52(3,149) | 0.04 | 0.02 | .076 | -0.04 | 0.01 | .006 | 0.00 | 0.01 | .791 |
| Volume | 2.62(3,149) | -0.58 | 2.99 | .846 | 2.38 | 1.70 | .164 | 2.77 | 1.16 | .018 |
| **SNC 8** | | | | | | | | | | |
| GA | 0.83(3,149) | -0.09 | 0.12 | .461 | 0.07 | 0.07 | .316 | 0.02 | 0.05 | .714 |
| Sex | 4.14(3,149) | 1.05 | 0.56 | .064 | 0.40 | 0.32 | .215 | -0.55 | 0.22 | .013 |
| PMA | 1.75(3,149) | 0.10 | 0.22 | .652 | -0.18 | 0.13 | .154 | -0.11 | 0.09 | .196 |
| IMD | 3.48(3,149) | 0.04 | 0.02 | .068 | -0.04 | 0.01 | .007 | 0.00 | 0.01 | .913 |
| Volume | 0.81(3,149) | -4.30 | 4.10 | .297 | 0.81 | 2.35 | .732 | -1.93 | 1.61 | .234 |
| **SNC 9** | | | | | | | | | | |
| GA | 0.85(3,149) | -0.07 | 0.12 | .561 | 0.06 | 0.07 | .378 | 0.01 | 0.05 | .810 |
| Sex | 4.14(3,149) | 1.03 | 0.56 | .065 | 0.40 | 0.32 | .211 | -0.56 | 0.22 | .013 |
| PMA | 1.75(3,149) | -0.01 | 0.20 | .960 | -0.16 | 0.11 | .163 | -0.16 | 0.08 | .047 |
| IMD | 3.55(3,149) | 0.04 | 0.02 | .064 | -0.04 | 0.01 | .007 | 0.00 | 0.01 | .859 |
| Volume | 1.54(3,149) | -5.97 | 3.36 | .078 | 2.16 | 1.94 | .266 | 0.49 | 1.34 | .717 |
| **SNC 10** | | | | | | | | | | |
| GA | 0.84(3,149) | -0.03 | 0.13 | .795 | 0.06 | 0.07 | .412 | 0.02 | 0.05 | .664 |
| Sex | 4.13(3,149) | 0.89 | 0.56 | .112 | 0.43 | 0.32 | .185 | -0.57 | 0.22 | .011 |
| PMA | 1.74(3,149) | 0.07 | 0.20 | .724 | -0.18 | 0.12 | .129 | -0.15 | 0.08 | .062 |
| IMD | 3.54(3,149) | 0.05 | 0.02 | .059 | -0.04 | 0.01 | .007 | 0.00 | 0.01 | .890 |
| Volume | 1.86(3,149) | -7.91 | 3.58 | .029 | 1.54 | 2.08 | .461 | -0.92 | 1.43 | .523 |
| **SNC 11** | | | | | | | | | | |
| GA | 0.86(3,149) | -0.09 | 0.13 | .481 | 0.06 | 0.07 | .382 | -0.01 | 0.05 | .844 |
| Sex | 4.22(3,149) | 1.05 | 0.56 | .065 | 0.39 | 0.32 | .227 | -0.59 | 0.22 | .007 |
| PMA | 1.83(3,149) | 0.02 | 0.21 | .920 | -0.18 | 0.12 | .120 | -0.21 | 0.08 | .010 |
| IMD | 3.50(3,149) | 0.04 | 0.02 | .072 | -0.04 | 0.01 | .006 | 0.00 | 0.01 | .710 |
| Volume | 2.83(3,149) | -1.84 | 3.87 | .634 | 1.71 | 2.21 | .441 | 4.06 | 1.49 | .007 |
| **SNC 12** | | | | | | | | | | |
| GA | 0.91(3,149) | -0.03 | 0.13 | .821 | 0.03 | 0.07 | .695 | 0.00 | 0.05 | .961 |
| Sex | 4.15(3,149) | 1.01 | 0.55 | .070 | 0.42 | 0.31 | .188 | -0.55 | 0.22 | .013 |
| PMA | 1.81(3,149) | 0.01 | 0.20 | .977 | -0.17 | 0.11 | .138 | -0.16 | 0.08 | .043 |
| IMD | 3.76(3,149) | 0.04 | 0.02 | .136 | -0.03 | 0.01 | .017 | 0.00 | 0.01 | .991 |
| Volume | **4.92(3,149)** * | -8.15 | 3.53 | .022 | 5.12 | 2.01 | .012 | 1.90 | 1.41 | .180 |
| **SNC 13** | | | | | | | | | | |
| GA | 0.84(3,149) | -0.06 | 0.12 | .641 | 0.06 | 0.07 | .377 | 0.01 | 0.05 | .787 |
| Sex | 4.14(3,149) | 1.06 | 0.56 | .058 | 0.40 | 0.32 | .219 | -0.56 | 0.22 | .013 |
| PMA | 1.74(3,149) | 0.06 | 0.20 | .773 | -0.18 | 0.12 | .131 | -0.16 | 0.08 | .048 |
| IMD | 3.52(3,149) | 0.04 | 0.02 | .110 | -0.04 | 0.01 | .010 | 0.00 | 0.01 | .876 |
| Volume | 1.27(3,149) | -8.28 | 4.54 | .070 | 1.82 | 2.62 | .487 | 0.13 | 1.81 | .944 |

| | | | | | | | | | |
|---|---|---|---|---|---|---|---|---|---|
| **SNC 14** | | | | | | | | | |
| GA | 0.85(3,149) | -0.09 | 0.12 | .473 | 0.07 | 0.07 | .320 | 0.01 | 0.05 | .823 |
| Sex | 4.18(3,149) | 1.12 | 0.56 | .048 | 0.39 | 0.32 | .234 | -0.58 | 0.22 | .009 |
| PMA | 1.76(3,149) | 0.05 | 0.20 | .793 | -0.17 | 0.12 | .136 | -0.17 | 0.08 | .030 |
| IMD | 3.51(3,149) | 0.04 | 0.02 | .129 | -0.04 | 0.01 | .011 | 0.00 | 0.01 | .984 |
| Volume | 1.50(3,149) | -7.74 | 4.90 | .116 | 1.56 | 2.82 | .581 | 2.25 | 1.93 | .246 |
| **SNC 15** | | | | | | | | | | |
| GA | 0.86(3,149) | -0.07 | 0.13 | .572 | 0.04 | 0.07 | .588 | 0.01 | 0.05 | .829 |
| Sex | 4.13(3,149) | 0.98 | 0.56 | .083 | 0.46 | 0.32 | .145 | -0.55 | 0.22 | .014 |
| PMA | 1.76(3,149) | 0.04 | 0.20 | .853 | -0.21 | 0.11 | .068 | -0.16 | 0.08 | .043 |
| IMD | 3.63(3,149) | 0.04 | 0.02 | .073 | -0.04 | 0.01 | .006 | 0.00 | 0.01 | .866 |
| Volume | 2.37(3,149) | -5.03 | 4.52 | .267 | 6.10 | 2.55 | .018 | 0.59 | 1.79 | .743 |
| **Sensitivity analyses (reporting SCN 12 only)** | | | | | | | | | | |
| **No twins/ triplets** | | | | | | | | | | |
| GA | 0.50(1,132) | -0.04 | 0.13 | .740 | -0.02 | 0.07 | .786 | -0.01 | 0.05 | .876 |
| Sex | 3.49(1,132) | 1.07 | 0.59 | .072 | 0.37 | 0.32 | .252 | -0.63 | 0.23 | .006 |
| PMA | 1.46(1,132) | 0.14 | 0.21 | .506 | -0.17 | 0.11 | .129 | -0.13 | 0.08 | .102 |
| IMD | 3.57(1,132) | 0.05 | 0.03 | .056 | -0.03 | 0.01 | .041 | 0.00 | 0.01 | .876 |
| Volume | **5.95(1,132)** | -9.73 | 3.77 | .011 | 5.58 | 2.03 | .007 | 2.21 | 1.44 | .128 |
| **No outliers** | | | | | | | | | | |
| GA | 0.49(1,132) | 0.02 | 0.11 | .892 | 0.00 | 0.08 | .973 | -0.03 | 0.05 | .549 |
| Sex | 4.16(1,132) | 0.59 | 0.48 | .223 | 0.42 | 0.33 | .203 | -0.48 | 0.21 | .025 |
| PMA | 1.46(1,132) | -0.05 | 0.17 | .779 | -0.18 | 0.12 | .132 | -0.23 | 0.08 | .004 |
| IMD | 3.57(1,132) | 0.06 | 0.02 | .008 | -0.03 | 0.01 | .015 | -0.01 | 0.01 | .535 |
| Volume | **5.95(1,132)** | -7.24 | 3.20 | .025 | 5.10 | 2.18 | .020 | 1.46 | 1.41 | .303 |
| **Controlling for lesions** | | | | | | | | | | |
| GA | 0.87(1,149) | -0.03 | 0.13 | .800 | 0.04 | 0.07 | .620 | -0.01 | 0.05 | .912 |
| Sex | 4.19(1,149) | 0.99 | 0.56 | .079 | 0.47 | 0.31 | .140 | -0.57 | 0.22 | .010 |
| PMA | 1.79(1,149) | 0.00 | 0.20 | .987 | -0.15 | 0.11 | .186 | -0.17 | 0.08 | .035 |
| IMD | 0.93(1,149) | 0.04 | 0.02 | .129 | -0.03 | 0.01 | .012 | 0.00 | 0.01 | .930 |
| Lesions | 5.76(1,149) | -0.25 | 0.56 | .655 | 0.52 | 0.32 | .103 | -0.22 | 0.22 | .333 |
| Volume | **0.87(1,149)** | -8.18 | 3.54 | .022 | 5.18 | 2.00 | .011 | 1.87 | 1.41 | .185 |
| **Controlling for maternal age and education** | | | | | | | | | | |
| GA | 0.88(1,146) | -0.02 | 0.12 | .877 | 0.01 | 0.07 | .944 | -0.01 | 0.05 | .909 |
| Sex | 4.34(1,146) | 0.89 | 0.54 | .100 | 0.57 | 0.32 | .074 | -0.53 | 0.22 | .016 |
| PMA | 1.80(1,146) | 0.02 | 0.19 | .912 | -0.12 | 0.11 | .310 | -0.17 | 0.08 | .032 |
| Maternal | 1.98(1,146) | | | | | | | -1.16 | 0.57 | .043 |
| education | | -1.91 | 1.40 | .176 | -0.19 | 0.83 | .823 | -0.81 | 0.50 | .110 |
| 17-19 | | -2.94 | 1.23 | .018 | 0.35 | 0.73 | .628 | -1.15 | 0.93 | .218 |

| | | | | | | | | | | |
|---|---|---|---|---|---|---|---|---|---|---|
| ≥19 Still in education | | -2.45 | 2.29 | .285 | 1.12 | 1.36 | .411 | | | |
| Maternal age | 3.22(1,146) | -0.11 | 0.05 | .028 | -0.06 | 0.03 | .038 | 0.01 | 0.02 | .783 |
| Volume | **4.63(1,146)** | -5.80 | 3.53 | .102 | 6.04 | 2.09 | .004 | 2.23 | 1.43 | .123 |

*Table S4.* Results of multivariate and univariate regression analyses of cognitively stimulating parenting on childhood outcomes (main analysis and sensitivity analyses)

| | | *Univariate* | | | | | | | | |
|---|---|---|---|---|---|---|---|---|---|---|
| | | ***PC1*** | | | ***PC2*** | | | ***PC3*** | | |
| *Predictor* | *Multivariate F(df)* | *β* | *SE* | *p* | *β* | *SE* | *p* | *β* | *SE* | *p* |
| **Main analysis** | | | | | | | | | | |
| GA | **2.79**(3,198)* | -0.08 | 0.10 | .450 | 0.16 | 0.06 | **.009** | 0.05 | 0.04 | .276 |
| Sex | **4.32**(3,198)* | 0.92 | 0.45 | .050 | 0.34 | 0.29 | .240 | -0.58 | 0.20 | **.004** |
| IMD | **5.50** (3,198)* | 0.03 | 0.20 | .102 | -0.04 | 0.01 | **.003** | -0.01 | 0.01 | .017 |
| Cog. stim. parenting | **5.02**(3,198)* | -0.34 | 0.10 | **<.001** | -0.00 | 0.06 | .967 | -0.08 | 0.04 | .059 |
| **Sensitivity analyses** | | | | | | | | | | |
| **No twins/ triplets** | | | | | | | | | | |
| GA | 2.42(3,167) | -0.14 | 0.11 | .198 | 0.15 | 0.06 | .021 | 0.05 | 0.05 | .297 |
| Sex | **3.81**(3,167)* | 1.07 | 0.53 | .047 | 0.31 | 0.29 | .300 | -0.59 | 0.22 | **.008** |
| IMD | **3.96** (3,167)* | 0.04 | 0.02 | .102 | -0.03 | 0.01 | .017 | -0.01 | 0.00 | .250 |
| Cog. stim. parenting | **3.57**(3,167)* | -0.34 | 0.11 | **.002** | 0.00 | 0.06 | .933 | -0.05 | 0.05 | .276 |
| **No outliers** | | | | | | | | | | |
| GA | 2.21(3,188) | -0.06 | 0.09 | .526 | 0.15 | 0.06 | .018 | 0.03 | 0.04 | .536 |
| Sex | **3.22**(3,188)* | 0.60 | 0.41 | .142 | 0.26 | 0.29 | .380 | -0.47 | 0.19 | .014 |
| IMD | **6.45**(1,188)* | 0.03 | 0.17 | .064 | -0.04 | 0.01 | **.002** | -0.01 | 0.00 | .089 |
| Cog. stim. parenting | **6.80**(3,188)* | -0.39 | 0.10 | **<.001** | 0.04 | 0.07 | .520 | -0.11 | 0.04 | **.015** |
| **Controlling for lesions** | | | | | | | | | | |
| GA | **2.87**(3,196)* | -0.08 | 0.10 | .462 | 0.16 | 0.06 | **.007** | 0.04 | 0.04 | .299 |
| Sex | **4.30**(3,196)* | 0.88 | 0.48 | .068 | 0.39 | 0.28 | .169 | -0.59 | 0.20 | **.003** |
| IMD | **5.67**(3,196)* | 0.03 | 0.02 | .103 | -0.04 | 0.01 | **.002** | -0.01 | 0.01 | .135 |
| Lesions | 1.92(3,196) | | | | | | | | | |
| Minor | | -0.24 | 0.51 | .631 | 0.52 | 0.30 | .084 | -0.20 | 0.21 | .349 |
| Major | | 1.45 | 0.98 | .143 | -0.81 | 0.58 | .164 | -0.03 | 0.41 | .942 |
| Cog. stim. parenting | **5.06**(3,196)* | -0.34 | 0.10 | **<.001** | 0.00 | 0.06 | .993 | -0.08 | 0.04 | .054 |
| **Controlling for maternal age and education** | | | | | | | | | | |
| GA | 2.61(3,195) | -0.07 | 0.10 | .509 | 0.16 | 0.06 | **.013** | 0.04 | 0.04 | .310 |
| Sex | **4.51**(3,195)* | 0.75 | 0.46 | .106 | 0.44 | 0.29 | .132 | -0.51 | 0.20 | **.010** |

| | | | | | | | | | | |
|---|---|---|---|---|---|---|---|---|---|---|
| Maternal age | **543**(3,195)* | -0.12 | 0.04 | **.003** | -0.05 | 0.03 | .041 | 0.01 | 0.02 | .396 |
| Maternal education | **1.96**(3,195)* | | | | | | | | | |
| 17-19 | | -1.99 | 1.14 | .084 | 0.31 | 0.71 | .660 | -0.81 | 0.49 | .098 |
| ≥19 | | -2.54 | 0.99 | **.011** | 0.66 | 0.62 | .283 | -0.46 | 0.42 | .275 |
| Still in education | | -2.68 | 1.91 | .162 | 1.15 | 1.19 | .336 | -1.09 | 0.81 | .181 |
| Cog. stim. Parenting | **3.68**(3,195)* | -0.26 | 0.10 | **.010** | 0.04 | 0.06 | .496 | -0.08 | 0.04 | .054 |

**References**


1. Makropoulos A, Gousias IS, Ledig C, Aljabar P, Serag A, Hajnal JV, et al. (2014): Automatic whole brain MRI segmentation of the developing neonatal brain. *IEEE transactions on medical imaging*. 33:1818-1831.
2. Avants BB, Epstein CL, Grossman M, Gee JC (2008): Symmetric diffeomorphic image registration with cross-correlation: evaluating automated labeling of elderly and neurodegenerative brain. *Medical image analysis*. 12:26-41.
3. Makropoulos A, Robinson EC, Schuh A, Wright R, Fitzgibbon S, Bozek J, et al. (2018): The developing human connectome project: A minimal processing pipeline for neonatal cortical surface reconstruction. *Neuroimage*. 173:88-112.
4. Avants B, Gee JC (2004): Geodesic estimation for large deformation anatomical shape averaging and interpolation. *Neuroimage*. 23:S139-S150.
5. Shi F, Yap P-T, Wu G, Jia H, Gilmore JH, Lin W, et al. (2011): Infant brain atlases from neonates to 1-and 2-year-olds. *PloS one*. 6:e18746.
6. Tzourio-Mazoyer N, Landeau B, Papathanassiou D, Crivello F, Etard O, Delcroix N, et al. (2002): Automated anatomical labeling of activations in SPM using a macroscopic anatomical parcellation of the MNI MRI single-subject brain. *Neuroimage*. 15:273-289.
7. Wolke D, Jaekel J, Hall J, Baumann N (2013): Effects of sensitive parenting on the academic resilience of very preterm and very low birth weight adolescents. *Journal of Adolescent Health*. 53:642-647.
8. Caldwell BM, Bradley RH (1984): *Home observation for measurement of the environment*. University of Arkansas at Little Rock Little Rock.
9. Sotiras A, Resnick SM, Davatzikos C (2015): Finding imaging patterns of structural covariance via non-negative matrix factorization. *Neuroimage*. 108:1-16.
